# Trends in research on sick sinus syndrome: A bibliometric analysis from 2000 to 2022

**DOI:** 10.3389/fcvm.2022.991503

**Published:** 2022-11-09

**Authors:** Xin’ai Zhang, Yong Zhao, Yutong Zhou, Jiayu Lv, Jiaran Peng, Haiyan Zhu, Ruxiu Liu

**Affiliations:** ^1^Department of Cardiovascular, Guang’anmen Hospital, China Academy of Chinese Medical Sciences, Beijing, China; ^2^Department of Cardiovascular, Dongzhimen Hospital, Beijing University of Chinese Medicine, Beijing, China

**Keywords:** sick sinus syndrome, sinus node dysfunction, bibliometric, CiteSpace, VOS viewer

## Abstract

Sick sinus syndrome (SSS) is a refractory arrhythmia disease caused by the pathological changes of sinoatrial node and its adjacent tissues. 2,251 publications related to SSS were retrieved from Web of Science database from 2000 to 2022 and analyzed by using VOS viewer and CiteSpace software. The results showed the United States dominated the field, followed by Japan, Germany, and China. SSS was closely related to risk factors such as atrial fibrillation and aging. Sick sinus syndrome, atrial fibrillation and sinus node dysfunction were the top three keywords that had the strongest correlation with the study. Pacemaker implantation, differentiation and mutation are research hotspots currently. Clinical studies on SSS found that sick sinus syndrome, atrial fibrillation, and pacemakers were the top three keywords that had the largest nodes and the highest frequency. In the field of basic applied research and basic research, atrial fibrillation and pacemaker cells were the focus of research. In conclusion, bibliometric analysis provided valuable information for the prevention, treatment and future research trends of SSS.

## Introduction

Sick sinus syndrome (SSS) is a group of syndromes in which the pacing and efferent functions of the sinoatrial node (SAN) are impaired due to organic changes in the SAN and its surrounding tissues, resulting in chronic arrhythmias with insufficient blood supply to the heart, brain, kidneys and other organs (1). SSS is usually diagnosed by the patient’s signs and symptoms examination as well as electrocardiogram examination. SSS is asymptomatic or mildly symptomatic in the early stages, and some patients with SSS are detected on physical examination. Manifestations of SSS mainly include sinus bradycardia, ectopic atrial bradycardia, sinus node outlet block, sinus arrest, sinus node arrest, brady-tachy arrhythmia syndrome, chronotropic insufficiency, and rhythmic separation, etc. (2). Supraventricular tachycardia occurs intermittently in about 50% of patients with SSS, which is known as brady-tachy arrhythmia syndrome, a typical clinical manifestation of SSS (3). Although SSS was closely associated with atrial fibrillation, the exact underlying pathogenesis remains unclear (4). In a human and animal models of atrial fibrillation, atrial fibrillation induced remodeling of the right and left atria (including the sinus node), leading to atrial fibrosis, impaired calcium channels, and abnormal gene expression in patients with SSS, which exacerbates sinus node dysfunction and the progression of atrial fibrillation (5–7). Other studies suggested that aging and myocardial fibrosis may be a common pathogenesis of SSS and atrial fibrillation, but the research could not account for all cases of SSS and more in-depth pathogenesis needs further research (8).

Impaired sinus node pacing with sinus bradycardia or sinus arrest, where the heart has difficulty compensating for the slowed or stopped heart rate with a reduction in cardiac output per boom, may result in signs and symptoms of cardiac insufficiency (9). The symptoms of SSS patients were gradually aggravated from mild fatigue, insomnia, memory loss, transient vertigo and syncope, which showed a positive correlation between the degree of organ ischemia and the duration of cardiac arrest (10). The DANPACE study identified the incidence, predictors and prognostic significance of syncope in 1415 patients with SSS, and after a mean follow-up of 5.4 years the results found a higher mortality rate in patients with SSS who experienced syncope (11). Epidemiological studies suggested that the age of onset of SSS is between the ages of 20 and 90 with an increasing incidence with age (12). SSS was most prevalent in the elderly, and its 5-year survival rate was 62−65%, and the incidence of embolism was 15% (13). The incidence of SSS has been increasing in United States since 2012, with more than 75,000 people suffering from SSS each year. Approximately, 1 in 600 patients with cardiovascular disease over 65 years old was a SSS patient (14). It was estimated that by 2060 there will be more than 172,000 new cases of SSS in the United States each year (15). Until now, artificial pacemaker implantation had been recognized as the most effective treatment for SSS, with more than 50% of surgical pacemaker implantations surgery worldwide are performed for SSS patients (16). However, there are still many patients for whom a pacemaker is not suitable and there is no evidence that many patients do have a reduction of mortality after implanting artificial pacemaker (17). In recent years, in order to overcome the drawbacks of traditional artificial pacemakers, more and more researchers have focused on biological pacemakers, and biological pacing treatment is becoming a research hotspot (18).

Bibliometrics had been recognized by researchers since 1958. It is a useful method to study the distribution of literature, quantitative relationships and regular changes by using mathematics, statistics and other measurement methods, which is often applied in libraries and information science schools (19). Scientific research often involves many interdisciplinary disciplines, and cross-nodes of related research fields can be found through bibliometrics analysis. The application of bibliometrics to the medical field helps researchers to quantitatively analyze the current status of research, publication trends, countries/regions, institutions, researchers, future research hotspots of a disease at the macro and micro levels. Bibliometrics was widely used for visualization of the development of research on a disease in recent years (20). Bibliometrics can provide useful information for the world’s scientific research and clinical workers, and also provide reliable standards for the standardization of academic quality and reference information for international academic exchange and cooperation. Currently, bibliometrics has been widely used in medical research fields, such as chronic heart failure, cardio-oncology, pulmonary hypertension, cardiomyopathy and cardiorenal syndrome (21).

Therefore, we collected qualitative and quantitative data of publications on SSS from 2000 to 2022 in the Web of Science core collection database (WoSCC, Clarivate Analytics, Philadelphia, PA, United States)^1^ and identified the most prolific countries, institutions, authors, journals, keywords, and research hotspots in the fields by using VOS viewer and CiteSpace. The objective of our study was to provide a comprehensive bibliometric analysis of SSS for the past 22 years worldwide, hoping to help scholars with the current status, research hotspots, and the future trends of SSS research. We hope that this study will have a positive effect on the development of research on SSS and provide new insight for researchers and clinicians working on SSS study.

## Materials and methods

### Data source and search strategy

Web of Science core collection, a web-based multidisciplinary literature database built by Thomson Reuters using the open environment of the Internet, was used as the data source for this study, and it was considered to be one of the most influential and authoritative databases in the world. The search parameters were set as follows: Subject term: [Sick Sinus Syndrome] or [Sinus Node Dysfunction]; Time span: [from 2000 to 2022]; Type of literature: [articles and reviews]; Citation indexes: [SCI-Expanded]; Language selection: [English]. All data searched were exported and saved in plain text format, including title, author, keywords, source, abstract and references. The search covered the period 01 January 2000 to 12 May 2022. The retrieval was conducted in 1 day (12 May 2022).

### Data retrieval and analysis

All research data were retrieved from WoSCC independently by two researchers (Yutong Zhou and Yong Zhao) to ensure the credibility, and imported into Microsoft Excel for data processing. The retrieved data were exported in plain text form. Firstly, we extracted indicators such as number of annual publications, countries/regions, source journals, institutions, authors, keywords, and frequency of citations from the retrieved data, and then analyzed these indicators statistically. To assess the scientific quality of the retrieved data, the impact factor (IF) and journal citation reports (JCR) categories of publications were extracted, and the IF and categories published by the JCR for 2021 were used for analysis in this study. IF is an evaluation metric for journals published by the JCR that represents the average number of citations per paper published in the journal in the last two years, which is an important indicator reflecting the academic level of the journal and the quality of the paper. JCR assigned each journal to its corresponding IF and ranks them by specific fields (Q1, Q2, Q3, and Q4). In addition, another indicator applied in this study was the h-index. It is also known as the h-factor, which is a new method to evaluate academic achievement. h stands for “high citations,” represented the number of h articles published by a researcher with each article receives at least h citations. For example, a researcher’s h-index is 20, which means that he has published at least 20 papers that have each been cited at least 20 times. Therefore, the h-index reflects a researcher’s scholarly status accurately. The higher a researcher’s h-index, the more influential his paper is. Therefore, it was often considered as a useful indicator to evaluate the scientific output and influence of a researcher, country or institution.

### Visualized analysis

**FIGURE 1:**
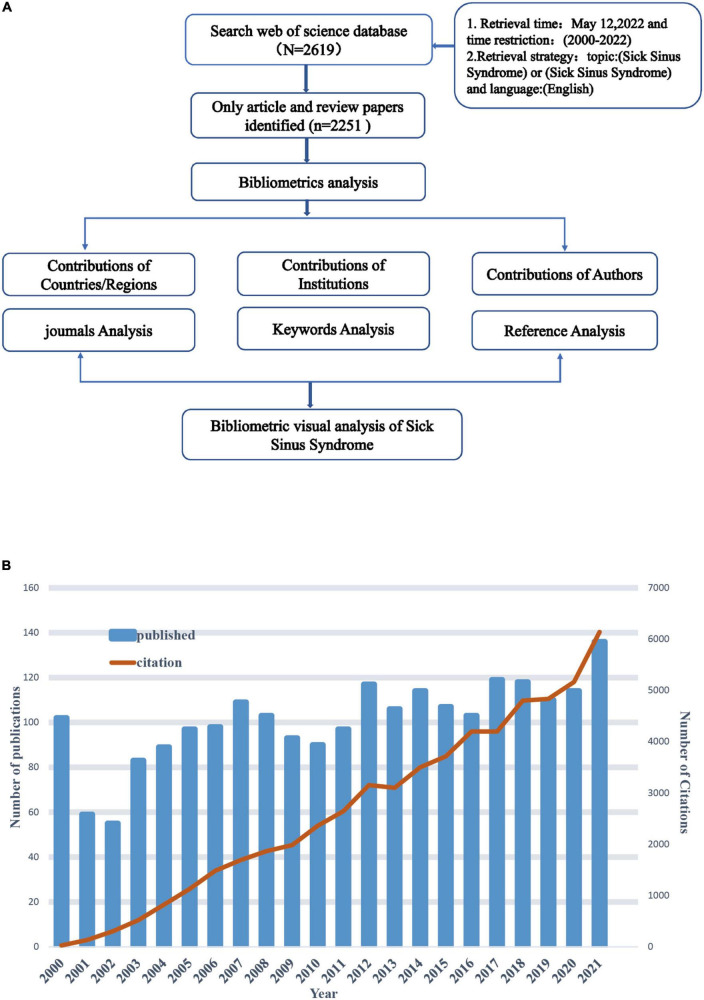
**(A)** Flow chart of literature retrieval of sick sinus syndrome (SSS) research. **(B)** Global trend chart of annual publications and citations related to SSS research from 2000 to 2022.

Network and density visualization maps were created by using VOS viewer version 1.6.18 (Technical Studies, Leiden University, Leiden, Netherlands) with nodes representing information such as times, countries/regions, institutions, authors, keywords, and cited references. The size of the nodes was determined by the weight of the elements, such as the number of publications, the number of citations or the frequency of occurrence. Each node was given a color, with the same color representing the same cluster. The links between nodes indicated the relevance of the elements to each other. The thickness of the links indicates the strength of the links, the thicker the link, the stronger the link. Total link strength (TLS) was adopted to quantitatively evaluate the links. Similar analysis was conducted to create visual maps of highly cited publications and the countries/regions, organizations, journals and authors of publications in each period to graph the evolution of these elements. CiteSpace V (Version 6.1 R2, Drexel University, United States) was employed to (i) create a visual map of the cited reference network from a time zone view, and (ii) detect a burst analysis of co-occurrence keywords. The parameters of CiteSpace were set as follows: time slice (2000−2022), year per slice (1), item source (all selected), node type (1 at a time), selection criteria (50), pruning (Pathfinder), visualization (cluster view static, showing the merged network). The detailed workflow for literature filtering and data analysis is shown in Figure 1A.

## Results

### Annual publication outputs and citation trend

The 2,619 publications were retrieved from WoSCC after the Subject terms were set as “Sick Sinus Syndrome” or “Sinus Node Dysfunction,” as well as the time span was set as “from 01 January 2000 to 12 May 2022.” A total of 2,251 publications of “articles” or “reviews” met the inclusion criteria for bibliometric analysis eliminating 176 conference abstracts, 135 editorial materials, 49 letters and 8 others. We can find from the chart of literature publications and citations that the horizontal coordinate represents the year, the left vertical coordinate represents the number of publications, and the right vertical coordinate represents the frequency of citations (Figure 1B). As can be seen from Figure 1B, the total citation frequency of publications were 59,399 times (49820 times, excluding self-citation), with an average of 2582.57 citations per year and 26.39 citations per paper. H-index was 105.

During 2000 to 2022, the annual number of publications (articles and reviews) was in the range of 30−70 with an upward trend overall, and the annual citation frequency of publications increased over time (Figure 1B). In terms of different stages: (i) 2000−2002: In this stage, the number of published papers per year showed a clear downward trend, and was reduced by half in 2002 compared with the number of papers published in 2000. The citations were in the range of 10−96, with a constant growth rate; (ii) 2002−2007: The number of papers published in this time period increased from 55 to 109 with a steady growth trend, followed by a short-term decline during 2008−2011. The growth rate of citations remained the same. (iii) 2010−2015: During which the number of publications on SSS in this time period showed a steady growth trend, followed by another short-term decline during 2015−2016. The growth rate of citations remained the same. (iv) 2016−2022: The number of publications keeps growing from 2016 to 2018, and then there was a slightly decrease in 2019. The number of papers continued to grow in the following 2 years and reached a growth peak in 2021. The growth rate of citations remained the same. Significantly, the output of publications in 2021 was 136, and had been cited for up to 6,138 times.

### Contributions of countries/regions

**FIGURE 2:**
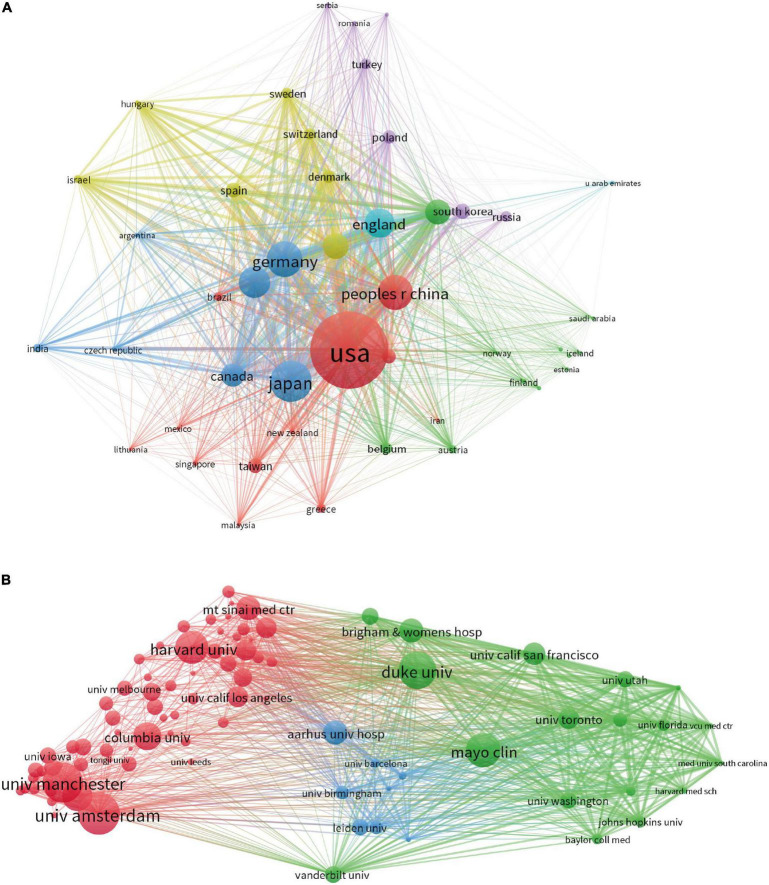
**(A)** Network map of Country/Region analysis based on VOS viewer. **(B)** Network map of Institutions analysis based on VOS viewer.

The 2,251 total publications (TP) were published by researchers in 77 countries/regions (Figure 2A). The top 10 countries accounted for 95.454% of the TP worldwide from 2000 to 2022 (Table 1). Among the top 10 countries in terms of TP, United States ranked first in terms of publications with 657 publications accounting for 29.14%, which was more than two times higher than Japan (271, 12.03%), who ranked second, and followed by Germany (222, 9.81%). For United States, both the total number of citations of total publications (TC = 25915) and the total link strength (TLS = 542392) were the highest. The other 7 countries in terms of TP are ranked as follows: China (214, 9.51%), Italy (189, 8.40%), England (169, 7.46%), France (143, 6.35%), Netherlands (139, 5.55%), Canada (125, 3.15%), and South Korea (71, 3.15%).

**TABLE 1:** The top 10 productive Country/Region of sick sinus syndrome.

Rank	Country	TP	Percentage (n/2251)	TC	CCP	TLS
1	UNITED STATES	656	29.14%	25915	39.50	542392
2	JAPAN	271	12.03%	3831	14.14	151326
3	GERMANY	221	9.81%	8698	39.36	268710
4	PEOPLES CHINA	214	9.51%	3189	14.90	166891
5	ITALY	189	8.40%	6310	33.39	255938
6	ENGLAND	168	7.46%	4946	29.44	277009
7	FRANCE	143	6.35%	5707	39.91	221597
8	NETHERLANDS	138	6.13%	6905	50.04	210964
9	CANADA	125	5.55%	7094	56.75	212764
10	SOUTH KOREA	71	3.15%	1063	14.97	45089

TP, total number of publications; TC, total number of citations of total publications; TLS, total link strength; CPP = TC/TP.

### Contributions of institutions

A total of 2,251 papers (TP) were contributed by 2,545 institutions (Figure 2B). Among the top ten prolific institutions presented in Table 2, seven institutions were from United States, while the other three institutions were from Netherlands, England, and Denmark. University of Amsterdam (TP = 47) was the most productive institution, followed by University of Manchester (TP = 45), Duke University (TP = 42), Mayo Clinic (TP = 39), Harvard University (TP = 37), Ohio State University (TP = 34), Columbia University (TP = 31), Aarhus University (TP = 27), Brigham Women S Hospital (TP = 27), Mount Sinai Medical Center (TP = 25). The top three institutions for total publications in terms of total citations and total contact strength were Harvard University (TC = 4004, TLS = 155), Duke University (TC = 3869, TLS = 226), and Brigham Women’s Hospital (TC = 3363, TLS = 154).

**TABLE 2:** The top 10 productive institutions of sick sinus syndrome.

Rank	Institutions	TP	TC	CCP	TLS	Countries
1	University of Amsterdam	47	3108	66.13	65	Dutch
2	University of Manchester	45	1639	36.42	61	England
3	Duke University	42	3869	92.12	226	United States
4	Mayo Clinic	39	1481	37.97	116	United States
5	Harvard University	37	4004	108.2	155	United States
6	Ohio State University	34	971	28.5	55	United States
7	Columbia University	31	915	29.52	64	United States
8	Aarhus University	27	766	28.37	47	Denmark
9	Brigham Women S Hospital	27	3363	124.5	154	United States
10	Mount Sinai Medical Center	25	1416	56.64	153	United States

TP, total number of publications; TC, total number of citations of total publications; TLS, total link strength; CPP = TC/TP.

### Authors and co-cited authors

**FIGURE 3:**
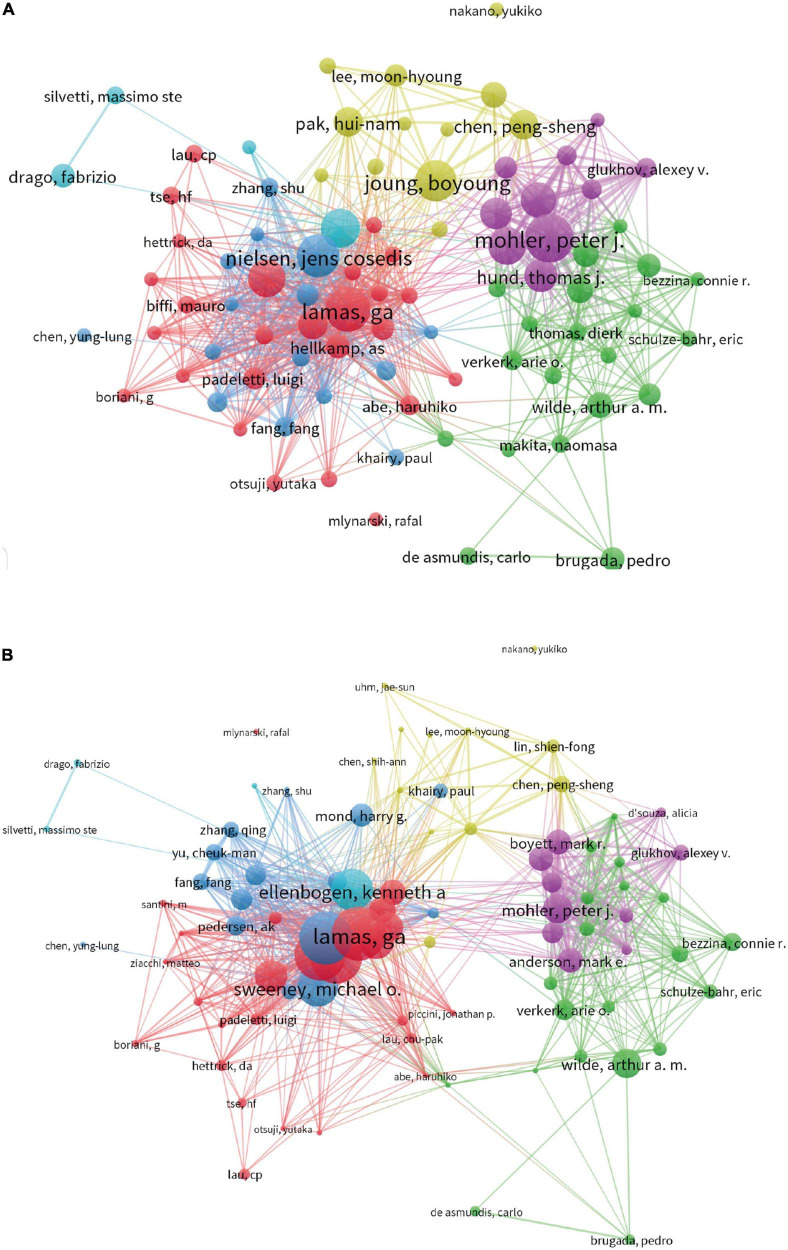
**(A)** Network map of authors analysis based on VOS viewer. **(B)** Network map of citation authors analysis based on VOS viewer.

A total of 2,251 papers were published by 10,746 researchers (Figures 3A,B). The top 10 most prolific authors and co-cited authors were presented in Table 3. Ellenbogen KA from the University of Liverpool ranked first with 35 publications (1.55%), followed by Lamas GA from Columbia University with 31 publications (1.38%), Nielsen JC from Aarhus University with 31 publications (1.38%), Boriani G from Universita di Modena e Reggio Emilia with 28 publications (1.24%), Mohler PJ from Ohio State University with 28 publications (1.24%), Dobrzynski H from Jagiellonian University with 25 publications (1.11%), Boyett MR from University of Liverpool with 22 publications (0.98%), Joung B from Yonsei University Health System with 21 publications (0.93%), Lee KL with 21 publications (0.93%), Sweeney MO from Truman Med Ctr with 21 publications (0.93%).

**TABLE 3:** The top 10 productive authors of sick sinus syndrome.

Rank	Authors	TP	Percentage (n/2251)	TLS	Institution
1	Ellenbogen KA	35	1.55%	32	University of Liverpool
2	Lamas GA	31	1.38%	66	Columbia University
3	Nielsen JC	31	1.38%	25	Aarhus University
4	Boriani G	28	1.24%	61	Universita di Modena e Reggio Emilia
5	Mohler PJ	28	1.24%	92	Ohio State University
6	Dobrzynski H	25	1.11%	59	Jagiellonian University
7	Boyett MR	22	0.98%	33	University of Liverpool
8	Joung B	21	0.93%	65	Yonsei University Health System
9	Lee KL	21	0.93%	54	None
10	Sweeney MO	21	0.93%	32	Truman Med Ctr

TP, total number of publications; TLS, total link strength.

### Journals and co-cited journals

**FIGURE 4:**
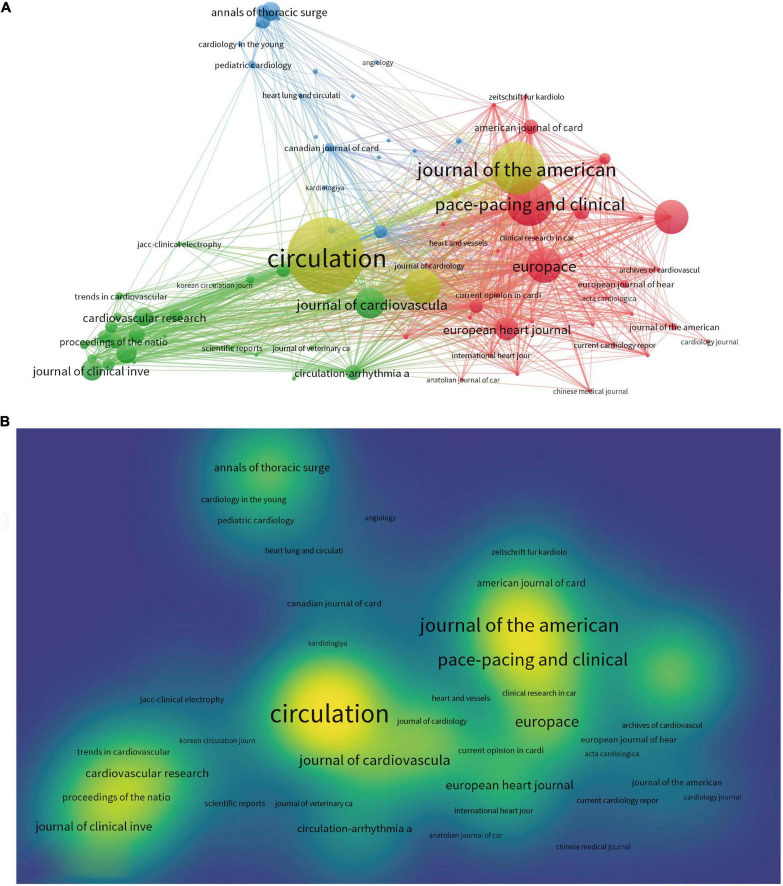
**(A)** Network map of journals analysis based on VOS viewer. **(B)** Density map of journals analysis based on VOS viewer.

A total of 2,251 papers (TP) were published in 542 different journals (Figures 4A,B). Table 4 presented the top 10 most published journals by name, volume, IF, JCR division, and publisher. *Pace Pacing and Clinical Electrophysiology*, the most prolific journal in terms of TP, was ranked first with 190 articles, accounting for approximately 32% of the TP. followed by *Europace* (133 articles, 5.91%, Q2), *Journal of Cardiovascular Electrophysiology* (82 articles, 3.64%, Q3), *Heart Rhythm* (71 articles, 3.15%, Q1), *Circulation* (54 articles, 2.4%, Q1), *Journal of Interventional Cardiac Electrophysiology* (51 articles, 2.27%, Q4), *International Journal of Cardiology* (41 articles, 1.82%, Q2), *Circulation Journal* (39 articles, 1.73%, Q3), *Journal of the American College of Cardiology* (37 articles, 1.64%, Q1), and *Annals of Thoracic Surgery* (36 articles, 1.60%, Q2).

**TABLE 4:** The top 10 productive journals of sick sinus syndrome.

Rank	Journal	TP	Percentage (%)	IF	Publishers	JCR
1	Pace Pacing and Clinical Electrophysiology	190	8.45%	1.976	Wiley	Q4
2	Europace	133	5.91%	5.214	Oxford Univ. Press	Q2
3	Journal of Cardiovascular Electrophysiology	82	3.64%	2.871	Wiley	Q3
4	Heart Rhythm	71	3.15%	6.343	Elsevier	Q1
5	Circulation	54	2.40%	29.69	Lippincott Williams and Wilkins	Q1
6	Journal of Interventional Cardiac Electrophysiology	51	2.27%	1.9	Springer	Q4
7	International Journal of Cardiology	41	1.82%	4.164	Elsevier	Q2
8	Circulation Journal	39	1.73%	2.993	Japanese Circulation Soc	Q3
9	Journal of the American College of Cardiology	37	1.64%	24.093	Elsevier	Q1
10	Annals of Thoracic Surgery	36	1.60%	4.33	Elsevier	Q2

TP, total number of publications; IF, impact factor; JCR, journal citation reports.

### References and co-cited references

As shown in Table 5, a total of 44238 references were cited in this study, which were mainly related to SSS, atrial fibrillation, heart failure, pacemaker implantation, differentiation, and molecular mechanisms (Table 5). The ten most-cited references were analyzed to reflect the current research status and research hotspots with a maximum IF of 91.25. Among the top 10 references, 9/10 were published in high impact journals including three were published in *New England Journal of Medicine*, five were published in *Circulation*, and one was published in *Journal of the American College of Cardiology*. Most of the papers were published in the field of “clinical trials, medical guidelines and basic experiments,” which may reflect that the research on SSS were mainly focus on clinical, therapeutic management and pathogenesis. The most cited article was a 6-year term prospective randomized controlled clinical trial on pacemaker therapy for sinus node dysfunction published in *Circulation* (Table 5). The article indicated that dual chamber pacing (DDDR) can better maintain AV synchronization compared with ventricular pacing in sinus node dysfunction (VVIR), consequently reducing the risk of hospitalization for heart failure and atrial fibrillation in SND patients (22). The second and third most cited article was a clinical practice guideline for device-based therapy of cardiac rhythm abnormalities published in *the New England Journal of Medicine* (23) and *Circulation* (24).

**TABLE 5:** The top 10 references of sick sinus syndrome.

Rank	References	First authors	Journal	IF	TC
1	Adverse effect of ventricular pacing on heart failure and atrial fibrillation among patients with normal baseline QRS duration in a clinical trial of pacemaker therapy for sinus node dysfunction.	Sweeney, MO	Circulation	29.69	1039
2	2012 ACCF/AHA/HRS Focused Update Incorporated Into the ACCF/AHA/HRS 2008 Guidelines for Device-Based Therapy of Cardiac Rhythm Abnormalities.	Epstein, AE	Journal of the American College of Cardiology	24.09	718
3	ACC/AHA/HRS 2008 Guidelines for Device-Based Therapy of Cardiac Rhythm Abnormalities A Report of the American College of Cardiology/American Heart Association Task Force on Practice Guidelines.	Epstein, AE	Circulation	29.69	647
4	Ventricular pacing or dual-chamber pacing for sinus-node dysfunction.	Lamas, GA	New England Journal of Medicine	91.25	639
5	Atrial high rate episodes detected by pacemaker diagnostics predict death and stroke Report of the atrial diagnostics ancillary study of the MOde Selection Trial (MOST).	Glotzer, TV	Circulation	29.69	534
6	The 11th World Survey of Cardiac Pacing and Implantable Cardioverter Defibrillators: Calendar Year 2009-A World Society of Arrhythmia’s Project.	Mond, Harry G	Pace-Pacing and Clinical Electrophysiology	1.976	521
7	Effects of physiologic pacing vs. ventricular pacing on the risk of stroke and death due to cardiovascular causes.	Connolly, SJ	New England Journal of Medicine	91.25	496
8	ACC/AHA/HRS 2008 Guidelines for Device-Based Therapy of Cardiac Rhythm Abnormalities.	Epstein, AE	Circulation	29.69	460
9	Electrical remodeling of the atria in congestive heart failure Electrophysiological and electroanatomic mapping in humans	Sanders, P	Circulation	29.69	441
10	Biventricular Pacing for Atrioventricular Block and Systolic Dysfunction.	Curtis, AB	New England Journal of Medicine	91.25	404

IF, impact factor; TC, total number of citations of total publications.

### Keyword analysis

**FIGURE 5:**
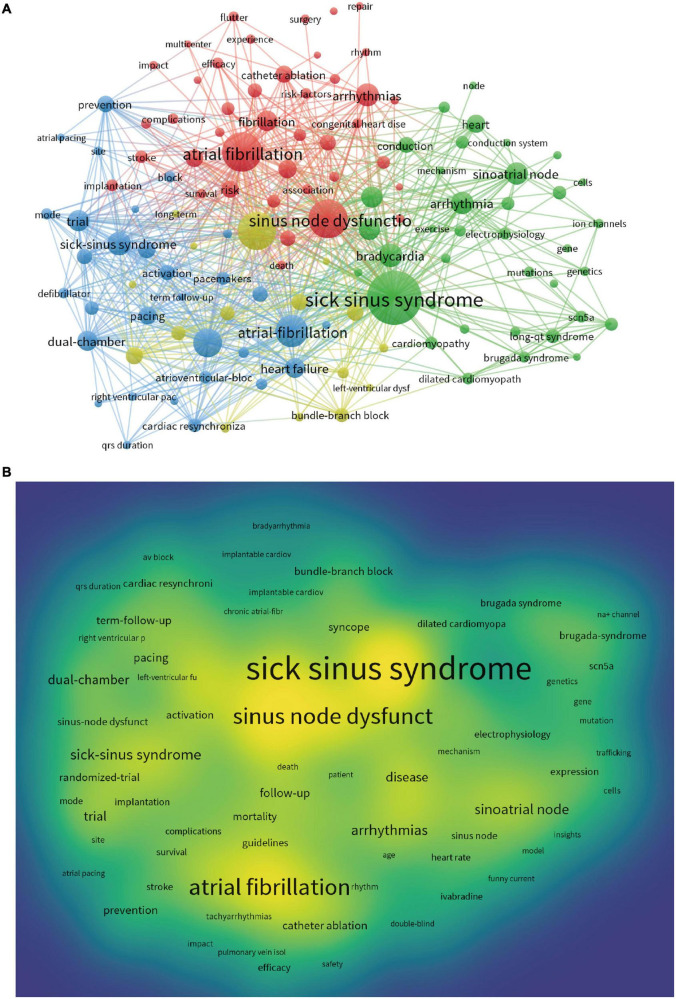
**(A)** Network map of keywords analysis based on VOS viewer. **(B)** Density map of keywords analysis based on VOS viewer.

**FIGURE 6:**
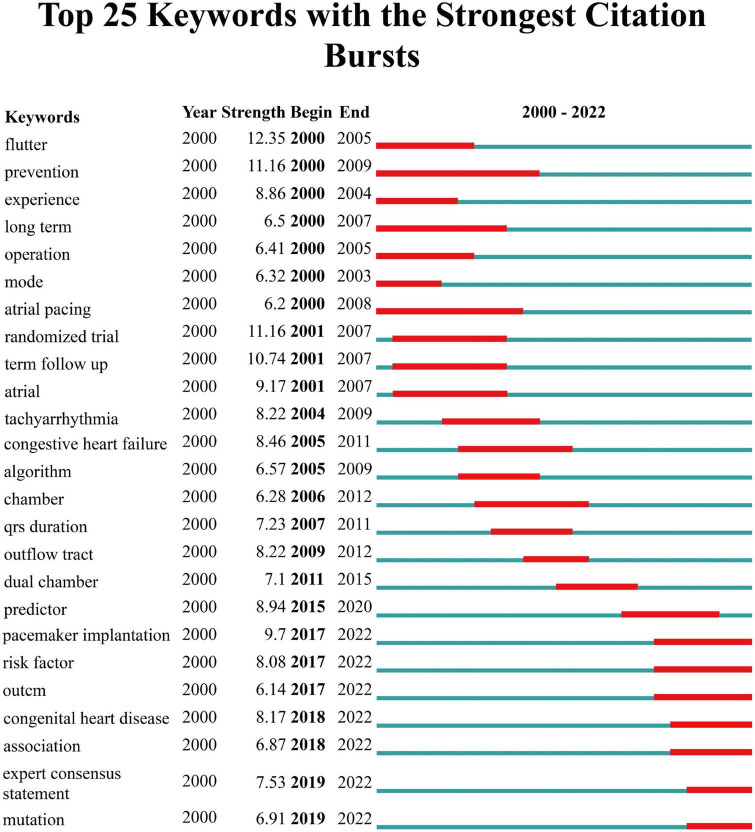
Top 25 Keywords of SSS with the strongest citation bursts based on CiteSpace.

We used VOS viewer to create a web map of keywords and the statistics showed that 6,447 keywords were extracted from 2,251 papers, of which 6 keywords appeared more than 200 times, 25 keywords appeared exceeding 100 times, and 74 keywords appeared more than 50 times (Figure 5A). Among the top 22 keywords listed in Table 6, the most frequently occurring keyword was sick sinus syndrome (538, TLS = 348), followed by atrial fibrillation (340, TLS = 307), sinus node dysfunction (334, TLS = 236), pacemaker (327, TLS = 311), atrial-fibrillation (258, TLS = 260), heart failure (216, TLS = 224), sinoatrial pacemaker cells (82, TLS 665) and aging (39, TLS 306). We analyzed the keyword co-linearity, clustering and density distribution, and used CiteSpace to plot the strongest citation bursts Figure 5B. From Figure 5A and Table 6, it can be seen that the three keywords sick sinus syndrome, atrial fibrillation and sinus node dysfunction had the largest nodes, the highest density and the highest frequency, and were the most relevant to the study of SSS, which is consistent with the topic of our study. Pacemaker implantation, heart-failure, differentiation and mutation were research hotspots currently (Figure 6). Notably, although the total amount of publications on the differentiation and mutation of SSS were relatively limited, it can be seen from the keyword bursts figure that the field of biological pacemaker had gradually become a research hotspot in recent years.

**TABLE 6:** The top 22 keywords in co-occurrence frequency of sick sinus syndrome.

Rank	Keyword	Co-occurrence	TLS
1	Sick sinus syndrome	538	2701
2	Atrial fibrillation	340	1976
3	Sinus node dysfunction	334	1695
4	Pacemaker	327	1870
5	Atrial-fibrillation	258	1544
6	Heart-failure	216	1379
7	Sick-sinus syndrome	164	1080
8	Bradycardia	164	1026
9	Arrhythmias	154	842
10	Sinoatrial node	153	791
11	Arrhythmia	148	806
12	Dysfunction	139	767
13	Trial	138	909
14	Disease	137	736
15	Fibrillation	135	795
16	Dual-chamber	130	911
17	Heart failure	129	921
18	Heart	115	527
19	Follow-up	113	693
20	Therapy	110	645
21	Sinoatrial pacemaker cells	82	665
22	Aging	39	306

TLS, total link strength.

### Pacemaker cells isolated from the sinoatrial node

**FIGURE 7:**
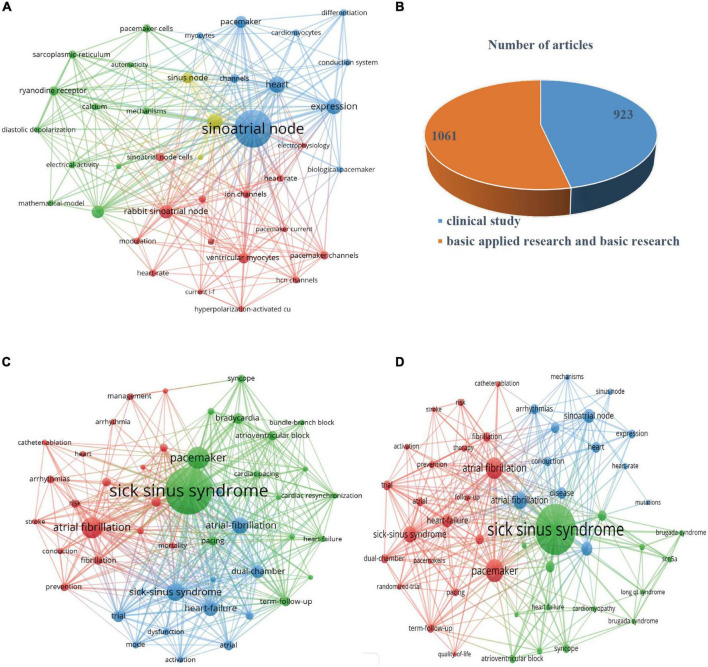
**(A)** Network map of pacemaker cells isolated from the sinoatrial node based on VOS viewer. **(B)** Numbers of articles of clinical research, basic applied research and basic research on SSS. **(C)** Network map of clinical research on SSS based on VOS viewer. **(D)** Network map of basic applied research and basic research on SSS based on VOS viewer.

Studying on pacemaker cells isolated from SAN is highly important for the research of SSS. Therefore, we conducted a secondary screening of the retrieved literature on SSS and identified 906 research papers (the type of literature was all articles) related to pacemaker cells isolated from SAN for statistical analysis and network diagrams production. As can be seen from the Figure 7A, most of the current research related to pacemaker cells isolated from the SAN was mainly basic research. The research hotspots were mainly focused on sinoatrial node, rabbit sinoatrial node, pacemaker activity, ryanodine receptor, ventricular myocytes, channels, ion channels, sarcoplasmic reticulum, funny current, calcium, electrical activity, etc. From the results of the statistical analysis, we can see that researchers are keen to investigate the principles of pacemaker cell operation and the mechanisms of lesions that cause pathological sinus node syndrome. However, we have not observed widespread clinical application of research results on pacemaker cells isolated from the SAN.

### Clinical, basic applied research and basic research of sick sinus syndrome

We conducted a secondary screening of the retrieved literature on SSS, and 1984 research papers were retrieved after the type of literature were set as “articles,” and the type of study were set as “clinical research” (923 articles) or “basic applied research and basic research” (1061 articles), (Figure 7B). We used VOS viewer to create a web map for statistical analysis (Figure 7C). And the statistics showed that the most frequently occurring keyword in clinical research was sick sinus syndrome (384, TLS = 2756), followed by atrial fibrillation (154, TLS = 1109), bradycardia (72, TLS = 924), term-follow-up (62, TLS = 778), and dual-chamber pacemaker (85, TLS = 773). As can be seen from Figure 7C, the pathogenesis of SSS was closely associated with atrial fibrillation and a lot of clinical trials have been conducted by researchers worldwide to study the relationship between atrial fibrillation and SSS. The most frequently occurring keyword in basic applied research and basic research was sick sinus syndrome (391, TLS = 1089), followed by pacemaker cells (15, TLS = 538), atrial fibrillation (149, TLS = 524). In the fields of basic applied research and basic research, pacemaker cells were the focus of research, involving studies on calcium clocks, ion currents, membrane clocks, and pacemaker-like cells (Figure 7D).

### Atrial fibrillation and sick sinus syndrome

**FIGURE 8:**
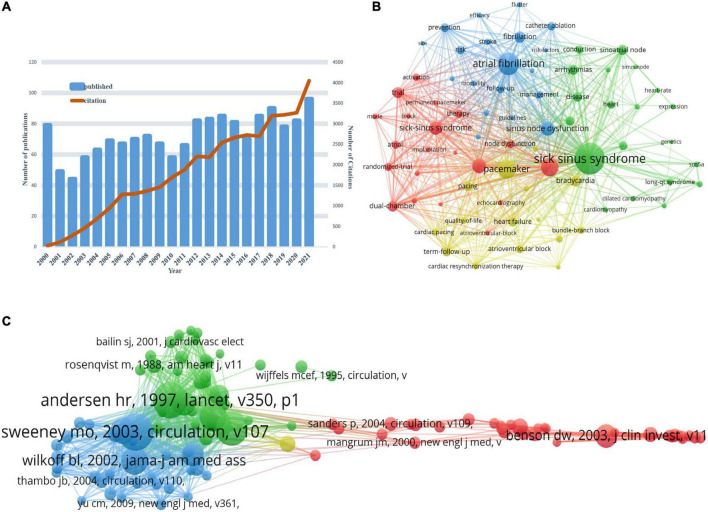
**(A)** Global trend chart of annual publications and citations of the study on the correlation between SSS and atrial fibrillation from 2000 to 2022. **(B)** Network map of the keywords of SSS and atrial fibrillation based on VOS viewer. **(C)** Network map of the references of SSS and atrial fibrillation based on VOS viewer.

We used VOS viewer to create a web map for the analysis of the correlation between SSS and atrial fibrillation (Figures 8B,C). We analyzed the number of publications, keyword co-linearity, clustering, references and co-cited references, and the statistics showed that 4,957 keywords were extracted from 1,627 papers, of which 4 keywords appeared more than 200 times, 16 keywords appeared exceeding 100 times, and 50 keywords appeared more than 50 times (Figures 8A–C). The most frequently occurring keyword was sick sinus syndrome (545, TLS = 1584), followed by atrial fibrillation (341, TLS = 1240), pacemaker (268, TLS = 1017), atrial-fibrillation (259, TLS = 908), heart failure (186, TLS = 826) and sinus node dysfunction (185, TLS = 679). From Figure 8B, it can be seen that the three keywords sick sinus syndrome, atrial fibrillation and pacemaker had the largest nodes, the highest density and the highest frequency. In addition, we analyzed the references and co-cited references of SSS and atrial fibrillation (Figure 8C). The most cited article was a review about the pathogenesis of atrial fibrillation published in *Nature*, in which the authors concluded that there is a clinical association between abnormal sinus node function and atrial fibrillation and that sinus node dysfunction and atrial fibrillation interact with each other (25). The second most cited article was a clinical trial paper published in *Circulation*, which found that ventricular desynchronization caused by ventricular pacing may increase the risk of hospitalization for heart failure and atrial fibrillation (22). The third one was a clinical guideline on the device therapy of cardiac rhythm abnormalities published in 2012 in *the New England Journal of Medicine* (23).

## Discussion

### Analysis of the current status of research

In terms of countries/regions, a country’s scientific research capacity was positively correlated with its economic power. The number of publications on SSS in United States were much more than that of other countries in the world, which means the United States was a world leader in this field. European countries were also stronger in the field of SSS study, with countries such as Germany, England, Italy, France, and Netherlands ranking in the top positions. Among Asian countries, Japan, China, and South Korea were relatively strong in SSS research. Countries in Africa and South America had fewer scientific outputs and were weaker in the field of SSS research. From the view of institutions, the institutions with the strongest research ability on SSS worldwide were University of Amsterdam, University of Manchester and Duke University. University of Amsterdam, founded in 1632, was one of the world’s leading research universities located in Amsterdam, with six Nobel Prize winners and five Dutch Prime Ministers produced here. The University of Manchester, a comprehensive university founded in 1824, was one of the top 50 universities in the world, with 25 Nobel Prize winners totally in the history. Duke University, founded in 1838, was one of the world’s leading research universities in the southern United States. Duke University was equally well known with MIT and the University of Pennsylvania.

Keywords was a significant indicator for reflecting the content of a study, and the frequency of a keyword may show the number of studies in a scientific field. In the keyword burst analysis from 2,251 publications on SSS, the frequency of occurrence of sick sinus syndrome, atrial fibrillation, sinus node dysfunction, and pacemaker were the highest, followed by pacemaker implantation, risk factors, heart disease, differentiation, and mutation in the last 3 years. Which indicated that the research on SSS was gradually intensifying, and more and more researchers had paid great attention to the risk factors and treatments for SSS (2). Up to date, a lot of scholars were devoting themselves to studying pacemakers, such as permanent pacemakers, pacemaker implantation methods, and complications management (26). And some scholars focused on the study of biological pacemakers, which may be a research hotspot on SSS in the future (27).

### Risk factors of sick sinus syndrome

The pathogenesis of SSS is related to many risk factors (28). A17-year term prospective study by Kristensen team found that the incidence of SSS increased with age, and the other risk factors mainly included atrial fibrillation, heart failure, obesity, NT-proBNP, hypertensive heart disease, gene mutations, and autonomic neuropathy, etc. (15, 29).

#### Aging

The function of the sinus node decreased with age, leading to an increase in the incidence of sinus node dysfunction and the number of pacemaker implantation in the elderly (15). Alghamdi et al. (30) assessed the potential mechanisms of aging and sinus node dysfunction, and found that aging-induced ion channel remodeling and abnormal Ca^2+^ handling produced bradycardia effects. Jones et al. (31) reported progressive loss of Cav1.2 protein in SAN tissues of aged guinea pig accompanied by reduced spontaneous activity. Zhang et al. (32) found upregulation of miR-1976 expression in plasma of age-related SSS patients. Interestingly, they further demonstrated experimentally that microRNA-1976 induces degeneration of the sinoatrial node by targeting and regulating Ca1.2 and Ca1.3 ion channels in rabbit sinoatrial node cells.

#### Atrial fibrillation

Atrial fibrillation and SSS often coexist, and they induce and perpetuate each other (33). More than 50% of patients with SSS may develop a brady-tachy arrhythmia syndrome, with tachyarrhythmias, most commonly atrial fibrillation and atrial flutter, leading to an increased risk of embolism and stroke (34). And anticoagulation therapy had been shown to be beneficial to reduce the risk of embolism and stroke (35). In addition, secondary sinus node dysfunction was associated with atrial tachyarrhythmias and atrial fibrillation. The incidence of sinus node dysfunction increased to 45% in patients with atrial fibrillation over 10 years or more (36). Pastore et al. (37) collected retrospective data from 313 patients implanted with dual-chamber pacemakers for sinus node dysfunction and found that the adoption of His bundle pacing reduced the risk of persistent atrial fibrillation in patients with sinus node dysfunction and long basal PR intervals.

#### Heart failure

Heart failure was a major risk factors for secondary sinus node dysfunction, the mechanism of which may be related to gene mutation in HCN4, a Na + /K + hyperpolarization-activated cyclic nucleotide-gated (HCN) channels that was a molecular marker of functional sinus node cell activity and played a key role in the pacemaker potential (38, 39). The HCN4 gene was closely related to various arrhythmias, and deletions or substitution mutations in exon bases of the HCN4 gene in patients with hereditary arrhythmias such as familial sinus bradycardia leading to alterations in the HCN4 channel protein, causing a significant reduction in the initiates the funny current and leading to a decrease in cardiac pacing function (40). Up-regulation of HCN4 gene expression in patients with acquired arrhythmias such as heart failure, cardiac hypertrophy, and atrial fibrillation increased the funny current and leaded to arrhythmias (28).

#### Others

The pathogenesis of SSS is associated with obesity, hypertensive heart disease, and autonomic neuropathy. Clapp et al. (41) found that obese patients with pacemaker placement were younger compared to non-obese patients with pacemaker implantation. Yanni et al. (42) found that obesity was associated with a function-dependent decrease in SAN and structural remodeling of the SAN, which includes SAN enlargement, SAN cells hypertrophy and extracellular matrix remodeling. Hypertensive heart disease was associated with sinus node dysfunction and reduced heart rate variability by a mechanism involving reduced responsiveness of SAN cells to angiotensin II agonists, which leads to sinus node dysfunction (43).

### Treatment strategies for sick sinus syndrome

#### Electrophysiological monitoring for sick sinus syndrome

According to *2018 ACC/AHA/HRS guideline on the evaluation and management of patients with bradycardia and cardiac conduction delay clinical guidelines*, prolonged ECG monitoring was recommended in patients with intermittent symptomatic bradycardia and conduction disturbances to correlate rhythm disorders with symptoms (44). A multi-centered study on the evaluation of syncope noted that extensive ECG abnormalities were associated with the increase of all-cause mortality at 1 year (45). It was appropriate for patients presenting with daily bradycardia and conduction disturbances to take 24−48 h ambulatory electrocardiogram monitoring (46). A study showed that when the heart rate comes within 40 bpm, the central blood pressure decreases, which means that the load on the target organs is lower, however, lower absolute central blood pressure values may be a sign of left ventricular dysfunction (47).

#### Drug treatment for sick sinus syndrome

Acute sinus node dysfunction was usually treated with medications or temporary transvenous or transesophageal pacing (48). Medications commonly used were (i) α/β agonists such as dopamine, epinephrine, and isoprenaline, which helped to increase the heart rate (49). However, dopamine and epinephrine may increase myocardial oxygen consumption, which were not suitable for SSS patients with ischemic cardiomyopathy (49). Isoprenaline was a non-selective beta agonist that could enhances sinus and atrioventricular node function. However, isoprenaline has the potential to predispose supraventricular tachycardia or beta receptor-dependent vasodilation, therefore it was usually used for acute sinus node dysfunction or electrophysiological assessment only (50). (ii) Muscarinic receptor inhibitors: Atropine was a muscarinic receptor inhibitor that was commonly used in the diagnostic valuation of sinus node dysfunction. Studies had shown an improvement in acute bradycardia and sinus node dysfunction with 0.5−2 mg doses of atropine (51). Atropine had been shown to be beneficial in SSS patients with combined hemodynamic instability, with minimal risk of worsening bradycardia, local ischemia or enhancing ventricular fibrillation (52). (iii) Adenosine receptor blockers: Aminophylline and theophylline extended-release tablets belonged to the methoxy anthracycline class and produced positive chronotropic action on the heart, probably mediated by inhibition of the inhibitory effect of adenosine on the sinoatrial node (53). (iv) Phosphodiesterase inhibitors: Cilostazol was used to prevent thrombosis. Several studies had shown that the drug had beneficial effects on heart rate in sinus node dysfunction patients with slow-fast syndrome, the mechanism of which may be related to an increase in sympathetic input due to systemic vasodilation and increased cAMP concentrations in sinus node pacemaker cells (54).

#### Electronic pacemaker implantation

Implanting electronic permanent pacemakers was the most effective treatment for reducing symptoms in SSS patients, and the number of pacemakers implanted was expected to double in the next 50 years (55). In a randomized study of symptomatic sinus node dysfunction patients who were randomized to an untreated group, an oral theophylline group, and a permanently paced group, the permanent pacemaker was superior in symptom control compared to the control group, despite the fact that theophylline increased resting heart rate (56). The ANSWER study, a prospective randomized single-blind controlled trial enrolling 650 patients at 43 study centers in Europe, evaluated the safety and efficacy of a conversion pacing mode conversion between single chamber atrial (AAI) pacing and dual chamber pacing (DDD) vs. a standard DDD pacing mode, and found a 51% reduction in the risk of death or hospitalization due to heart failure in patients with switching pacing modes after 3 years of follow-up (57). Temporary transvenous pacing can increase heartrate and improve symptoms in SSS patients with persistent hemodynamic instability and ineffective drug therapy (58). In patients with symptoms directly attributable to sinus node dysfunction, permanent pacing may increase the heart rate and improve symptoms of cerebral hypoperfusion caused by bradycardia (59).

### Prospects for research development in sick sinus syndrome

The application of electronic pacemakers had improved the quality of life for many patients with SSS, but it still has problems with infection, post-cardiac injury syndrome, wire loss and limited electronic life (60). In addition, many patients were not suitable or unwilling to undergo permanent pacemaker implantation (61). These problems limited the electronic pacemaker as the most ideal treatment for SSS, and scientists began to look for a more ideal treatment, therefore, the biological pacemaker was born on demand under such a background (62). The biological pacemaker-built sinus node pacing-like cells that perform normal pacing functions in the heart mainly through the regulation of single gene, multiple gene and signaling pathway (63). Current studies have found that genes and signaling pathways involved in biological pacing include T box gene 3 (Tbx3), Tbx 18, Insulin-like growth factor-1(ISL-1), short state homeobox 2 (Shox2), NK2 homeobox 5, Wnt signaling pathway and HCN signaling pathway, etc. (64, 65). At present, there are extensive studies on the genes of Tbx family, including Tbx 1, Tbx 3, Tbx 5, and Tbx 18 (66). Different genotypes played different roles in the development of the heart. Among which, Tbx 3 was not involved in the morphological development of the SAN but played an important role in regulating the function of the SAN (67). Tbx 18 gene was mainly involved in the development of the head of the SAN (68). ISL-1 was one of the molecular markers of undifferentiated cardiac histocytes/heart cells in the second cardiac region, which can promote cardiac development and differentiation (69). Study had found that mouse embryos with the ISL-1 gene knocked out had cardiac arrest or severe cardiac malformations at day 9.5 (70).

The Wnt signaling pathway was divided into the classical Wnt signaling pathway and the non-classical Wnt signaling pathway, with the former playing a positive regulatory role in the development of the SAN (71). The classical Wnt signaling pathway functioned by activating a combination of maturation markers specific to pacemaker CMs (Shox2, ISL-1, Tbx18, HCN 4, and Tbx3), such as the induction of cardiac mesoderm formation and specific expansion of cardiac progenitor cells (72). Activation of classical Wnt signaling in human induced pluripotent stem cells derived from cardiac progenitor cells can initiate a gene regulatory program that inhibits NK2 homeobox 5 expression and promotes synergistic expression of Shox2, ISL-1, Tbx3, and Tbx18, thereby promoting the differentiation of cardiac progenitor cells into human pacemaker-like cardiomyocytes capable of pacing human heart (73, 74). However, most of the research so far had been focused on basic experiments, and the efficiency of targeted differentiation of pacemaker-like cells through regulation of genes and signaling pathways was relatively low (75). In addition, there is also a risk of causing inflammatory reactions and cytotoxicity in the organism, and more research are still needed in the future to overcome these problems and to provide a scientific basis for the study of biological pacing in humans and its gradual clinical application (68).

## Conclusion

In this study, we summarized the current research status, countries/regions, authors, keywords, and research hotspots of SSS in the past 22 years with bibliometric visualization analysis, which revealed that the research for SSS were mainly focused on diagnostic, preventive, and clinical trials. Pacemaker implantation, atrial fibrillation, ischemic cardiomyopathy, differentiation, and gene mutations were potential research hotspots in recent years. In conclusion, bibliometric analysis provided a comprehensive review of research results in the field of SSS for researchers to grasp the research framework of SSS and find new perspectives for future research.

## Author contributions

XZ and YoZ designed the study. YuZ and JL collected the data. XZ and JP reviewed all the data. JP analyzed the data. XZ completed the manuscript. HZ and RL reviewed and revised the manuscript. All authors contributed to the article, approved the submitted version, and agreed to be responsible for all aspects of the work.

## Footnotes

1
https://access.clarivate.com/login?app=wos&alternative=true&shibShireURL=https:%2F%2Fwww.webofknowledge.com%2F%3Fauth%3DShibboleth&shibReturnURL=https:%2F%2Fwww.webofknowledge.com%2F&roaming=true
